# Development of a Highly Sensitive Nested PCR and Its Application for the Diagnosis of Cutaneous Leishmaniasis in Sri Lanka

**DOI:** 10.3390/microorganisms10050990

**Published:** 2022-05-09

**Authors:** Nirmitha Lalindi De Silva, Viraji Nefertiti Hiromel De Silva, Arachchige Theja Hemapala Deerasinghe, Upeksha Lakmini Rathnapala, Makoto Itoh, Hidekazu Takagi, Mirani Vasanthamala Weerasooriya, Hirotomo Kato, Thishan Channa Yahathugoda

**Affiliations:** 1 Department of Parasitology, Faculty of Medicine, University of Ruhuna, Galle 80000, Sri Lanka; lalindidesilva@med.ruh.ac.lk (N.L.D.S.); miraniweera@yahoo.co.uk (M.V.W.); tcyahath@med.ruh.ac.lk (T.C.Y.); 2 Base Hospital Tangalle, Tangalle 82200, Sri Lanka; virajine@gmail.com; 3 District General Hospital Hambantota, Hambantota 82000, Sri Lanka; athdeerasinghe@yahoo.com; 4 School of Biosciences, University of Melbourne, Melbourne, VIC 3010, Australia; gurathnapala@unimelb.edu.au; 5 Department of Microbiology and Immunology, Aichi Medical University School of Medicine, Aichi 480-1195, Japan; macfilaria@gmail.com (M.I.); htakagi@aichi-med-u.ac.jp (H.T.); 6 Division of Medical Zoology, Department of Infection and Immunity, Jichi Medical University, Tochigi 329-0498, Japan

**Keywords:** cutaneous leishmaniasis, ITS, PCR-RFLP, Sri Lanka

## Abstract

The recent surge in cutaneous leishmaniasis (CL) in Sri Lanka has rendered clinical diagnosis difficult; thus, laboratory confirmation is indispensable. A modified (two novel inner primers to detect CL caused by *Leishmania donovani*) nested Internal Transcribed Spacer-1 (ITS1) PCR-Restriction Fragment Length Polymorphism (RFLP) method was developed and tested. The sensitivity of the modified nested PCR was tested using serial dilutions (10^3^ to 10^−2^) of the DNA extract of a cultured *L. donovani* DD8 strain. Patients (*n* = 194) from Southern Sri Lanka were examined clinically, microscopically (Slit Skin Smear-SSS) and using the modified nested PCR. The modified nested PCR detected 2.55 fg of parasite DNA compared to ITS1 PCR (25 fg) and detected more cases than SSS (94.3% vs. 77.3%; *p* < 0.01). The RFLP pattern was *L. donovani* in all cases. The modified nested PCR performed well in clinically doubtful lesions (95% by PCR vs. 60% by SSS; *p* < 0.01), ulcerated nodules (91% vs. 71.8%; *p* < 0.01) and plaques (100% vs. 66.7%; *p* < 0.01). SSS demonstrated sensitivity (80.9%), specificity (81.8%), PPV (98.7%) and NPV (20.5%) against modified PCR. Low parasite loads and atypical lesions can be diagnosed by the proposed method with higher accuracy.

## 1. Introduction

Leishmaniasis is a parasitic disease that is classified as a neglected tropical disease. It is caused by a protozoan parasite belonging to the genus *Leishmania.* Over 20 species of the genus *Leishmania* are known to cause disease in humans [1]. The causative species is a key determinant of the clinical outcome, which may take the form of either cutaneous, mucocutaneous or visceral disease [2].

Sri Lanka has been a recent focus of leishmaniasis in the Indian subcontinent, which is currently experiencing a surge in the cutaneous form of the disease. The upsurge observed over the last decade led to leishmaniasis being listed as a notifiable disease in Sri Lanka in 2008 [3]. Annual case numbers in the early 2000s were low, with 22 cases reported in the year 2001 [4]. According to the Weekly Epidemiological Reports of the Epidemiology Unit of the Ministry of Health Sri Lanka, by 2017, there were 1470 cases, which showed a rapid increase up to 3964 and 3123 in 2019 and 2020, respectively. Despite attempts to identify a non-human reservoir host, the evidence generated so far has been insufficient to incriminate or exclude any animal reservoirs [5,6,7]. It was suggested that a human reservoir was likely [8]. According to published data, canine leishmaniasis does not appear widespread in Sri Lanka yet. Studies aiming to find animal reservoirs found 2 out of 151 dogs (1.32%) positive for *Leishmania* amastigotes [5], rK39 positivity in 1 out of 114 dogs (0.87%) [6], and in 4 out of 51 (7.8%) dogs in another study [7]. A more recent survey among 668 dogs in 18 out of 25 districts in Sri Lanka revealed a *Leishmania* amastigote prevalence of only 0.9% (2/668) in blood [9]. These findings suggest the possibility of dogs acting as reservoir hosts [10,11].

The dominant clinical form of leishmaniasis in Sri Lanka is cutaneous leishmaniasis (CL). The causative parasite was identified as *Leishmania donovani* zymodeme MON-37, a naturally attenuated form of the parasite. However, *L. donovani* causes visceral leishmaniasis (VL) in India and East Africa [12]. A recent study provided evidence that the local parasite’s genome had homologous polymorphisms to *L. tropica* and *L. major*, suggesting hybridization and probable origin and importation from East Africa [13]. A few cases of locally acquired VL (now considered an emerging infection in Sri Lanka) and mucocutaneous leishmaniasis were also reported [14,15]. 

In the absence of a separate anti-leishmania campaign, the rising case burden, the emergence of atypical clinical variants [16] and poor response to treatment [17], early diagnosis and treatment to reduce the human reservoir have become a priority health concern in the country. However, the diagnosis of CL in Sri Lanka still largely relies on clinical diagnosis followed by slit skin smear (SSS) microscopy, which is widely available and less costly. However, case detection with SSS is a challenging task due to its variable sensitivity, which relies on many factors, including the parasite load, sampling technique and processing and examination-related factors, of which the expertise of the examiner is a key determinant. 

Nucleic acid-based methods are highly sensitive and specific and are well established for the diagnosis of leishmaniasis and the identification of its vectors [18]. In Sri Lanka, CL can be diagnosed with an accuracy of 92% with both Internal Transcribed Spacer-1 (ITS1) PCR (*Leishmania* genus-specific) and kDNA-PCR (*L. donovani* species-specific) [19]. A more recent study described a modified version of a nested PCR (Mo-STNPCR) to be 100% sensitive and 100% specific in diagnosing CL in Sri Lanka [20]. Loop-mediated isothermal amplification assay (LAMP) assay, which is technically less demanding than PCR, detected the local parasite strain with a sensitivity and specificity of 82.6% and 100%, respectively [21]. The availability of these techniques remains limited to reference centers, of which none are situated in the southern focus of disease transmission. Although molecular diagnoses remain expensive and more technically demanding, they need to be made more widely available in order to enhance case detection, as missed cases contribute to onward transmission. Additionally, the PCR technique is indispensable for settings such as Sri Lanka, where other infectious diseases mimicking CL, such as cutaneous tuberculosis and leprosy, are also present.

Internal Transcribed Spacers (ITSs), which are noncoding regions of rRNA genes, are highly conserved among *Leishmania* species, and amplification of the ITS1 region that lies between genes coding for small-subunit RNA and 5.8S rRNA genes enables the direct identification of *Leishmania* parasites from clinical lesions [22,23]. ITS1 PCR is known to be especially useful for detecting old world *Leishmania* species [24]. LITSR/L5.8S primers amplify a 320 bp fragment of the ITS1 region of *Leishmania* genus-specific DNA [25]. Restriction Fragment Length Polymorphism (RFLP) enables the identification of the causative species by restriction digestion of the PCR product. PCR-RFLP has been used successfully to identify *Leishmania* parasites to the species level and is much less laborious than other methods used for species identification, such as gene sequencing, multilocus enzyme electrophoresis (MLEE) or isoenzyme analysis [24]. In the present study, we developed and tested a modified nested ITS1 PCR-RFLP assay with novel inner primers with enhanced sensitivity for the diagnosis of CL and identification of the causative species in Sri Lanka. We aimed to further improve the sensitivity of the classic LITSR/L5.8S primers beyond the 92% sensitivity (detection level of 10 fg) that they have demonstrated so far in Sri Lanka [19] for the diagnosis of CL. Improving the sensitivity and specificity of the existing techniques and making them available is imperative to enhance the diagnosis of cases that are missed by the SSS, which is the routine diagnostic technique. Early case detection and treatment is the mainstay of disease control in Sri Lanka. 

## 2. Materials and Methods

### 2.1. Study Area and Samples 

The study was conducted in the Hambantota District in Southern Sri Lanka. The area consists of flat low land and is vastly covered by dry savannah and paddy cultivation. Adult patients (aged 18 years or more) with skin lesions suggestive of CL and residing in the Hambantota District, presenting for the first time to the dermatology clinic of the selected study site (Base Hospital Tangalle, which is a government sector hospital operating under the Ministry of Health Sri Lanka), were recruited. Informed written consent was obtained from all participants and ethical approval was granted by the Ethics Review Committee of the Faculty of Medicine, University of Ruhuna, Sri Lanka (approval no; 19.09.2018:3.5). Children were excluded due to the invasive nature of the sample collection. The skin lesions were clinically evaluated by a dermatology specialist and they were categorized into two groups, clinically confirmed and doubtful. Slit skin smears and a 3 mm punch biopsy were collected from the lesions of 194 patients. Both samples were obtained from the same lesion. Patients with lesions on the face, close to the eyes, nasal cartilage or pinna, were excluded from the study as these sites were either cosmetically or medically unfavorable for a punch biopsy. Sample collection was carried out by trained medical officers of the dermatology clinic. 

### 2.2. Giemsa Stained Slit Skin Smears

Two slit skin smears (SSS) were prepared from each lesion and they were stained with Giemsa stain and were independently examined and reported by two study authors.

### 2.3. Molecular Diagnosis 

#### 2.3.1. Sensitivity of the Modified Nested ITS1 PCR

**Figure 1:**
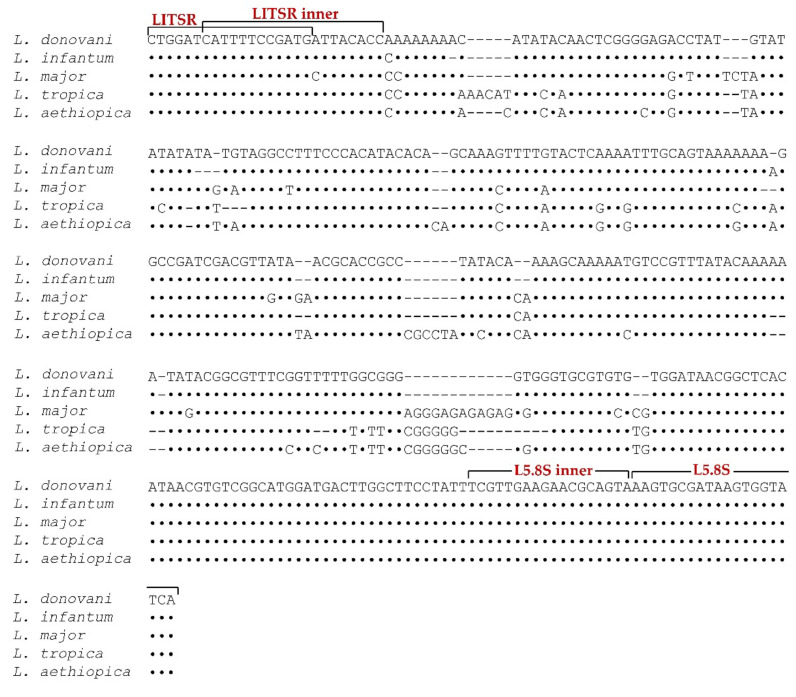
Alignment of Internal Transcribed Spacer 1 and 5.8S ribosomal RNA gene fragments of different *Leishmania* species. The dots denote identical sequences with those of *Leishmania donovani* and the dashes indicate gaps introduced for maximal alignment. The positions of the primers used in this study are marked.

The novel inner primers were designed based on the sequences conserved among *L. donovani*, *L. infantum*, *L. major*, *L. tropica*, and *L. aethiopica*. Figure 1 shows the alignment of the ITS1 and 5.8S ribosomal RNA gene fragments of different *Leishmania* species and the positions of the primers used in the present study. 

The sensitivity of ITS1 PCR and modified nested ITS1 PCR was assessed using the LITSR and L5.8S outer primers [25] and the novel inner primers (Table 1). Serial dilutions from 10^3^ to 10^−2^ were prepared from DNA extracted from cultured *L. donovani* DD8 strain using the QIAGEN DNeasy^®^ blood and tissue kit. PCR was carried out at each dilution, and the products were subjected to agarose gel electrophoresis. 

#### 2.3.2. DNA Extraction

Punch biopsy specimens stored in absolute ethanol were cut in half, and one half was used for DNA extraction. DNA was extracted using the QIAGEN DNeasy^®^ blood and tissue kit according to the manufacturer’s protocol. The extracted DNA was stored at −30 °C until used for molecular analysis. 

#### 2.3.3. Modified Nested ITS1 PCR 

The Internal Transcribed Spacer (ITS) region of *Leishmania* species was amplified using pairs of specific primers (Table 1). First, ITS1 PCR amplification was performed using a pair of outer primers, LITSR and L5.8S [25]. PCR amplification was performed in a total volume of 15 µL consisting of primers (0.3μMeach), KAPATaq ReadyMix 7.5 µL (KAPABIOSYSTEMS), and 0.5 µL of the template (Extracted DNA). Amplification was performed with 30 cycles of denaturation (95 °C for 1 min), annealing (55 °C for 1 min), and polymerization (72 °C for 1 min), followed by a final extension (72 °C for 7 min). A 0.5 μL portion of the PCR product was re-amplified using the pair of novel inner primers LITSR-inner and L5.8S-inner in the same manner as described above. 

#### 2.3.4. Restriction Fragment Length Polymorphism (RFLP) Analysis 

The nested PCR product was digested by the *Hae*III restriction enzyme, and the resulting restriction fragment patterns were analyzed by 2% agarose gel electrophoresis. The DNA size marker used was GeneRuler 100 bp Plus DNA Ladder (Thermo Fisher Scientific, Waltham, MA, USA). The gel was stained with GelRed Nucleic Acid Gel Stain (Biotium, Hayward, CA, USA), and DNA fragments were visualized with a UV transilluminator. *Hae*III restriction digestion and RFLP analysis were performed for *L. tropica*, *L. major*, and *L. donovani* DNA to obtain the specific restriction banding patterns that enable species identification.

### 2.4. Data Management and Statistical Analysis 

IBM SPSS Statistics Version 25 was used for data analysis. Sensitivity (SN), specificity (SP), positive predictive value (PPV), and negative predictive value (NPV) were estimated for the routine diagnostic method in comparison to nested ITS1 PCR. Detection rates of different lesions by SSS were compared with nested PCR by cross-tabulations, Pearson’s chi-square, or Fisher’s exact test. An approximate 2-sided 95% Confidence Interval (CI) was calculated for each proportion.

## 3. Results

### 3.1. Clinical Examination and Slit Skin Smear Microscopy 

Out of the 194 cases recruited for the study, the diagnosis was clinically confirmed by the dermatology specialist as CL in 175 cases (90.2%). However, the rest (*n* = 19 (9.8%)) presented with different clinical features that the dermatologist categorized as doubtful. In the present study, we categorized all the lesions under the following lesion types. The clinically doubtful lesions were also included and categorized under the most comparable lesion type. Papules (*n* = 23; 11.9%), nodules (*n* = 32; 16.5%), ulcerated nodules (*n* = 78; 40.2%), dry ulcers (*n* = 26; 13.4%), wet ulcers (*n* = 14; 7.2%), and plaques (*n* = 21; 10.8%) were the lesion types observed Smear positivity rates for investigator 1 and investigator 2 were 71.6% (*n* = 139/194) and 69.1% (*n* = 134/194), respectively, whereas the positive rate for any investigator was 77.3% (*n* = 150/194). 

### 3.2. Sensitivity of the Modified Nested ITS 1 PCR

**Figure 2:**
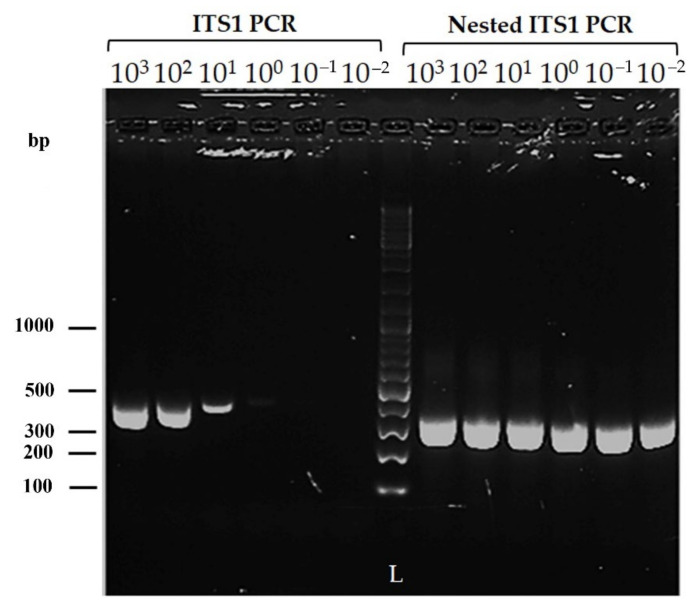
Sensitivity of ITS1 and modified nested PCR using *L. donovani* DD8 strain.

*Leishmania* genus-specific outer primers LITSR and L5.8S (Table 1) in ITS1 PCR detected up to 25 fg of parasite DNA at 10^−1^ dilution (Figure 2). The novel inner primers LITSR-inner and L5.8S-inner (Table 1) detected as little as 2.55 fg of parasite DNA (at 10^−2^ dilution: Figure 2).

### 3.3. Analysis of the Cohort of Cutaneous Leishmaniasis Cases by the Nested ITS1 PCR-RFLP (n = 194)

**Figure 3:**
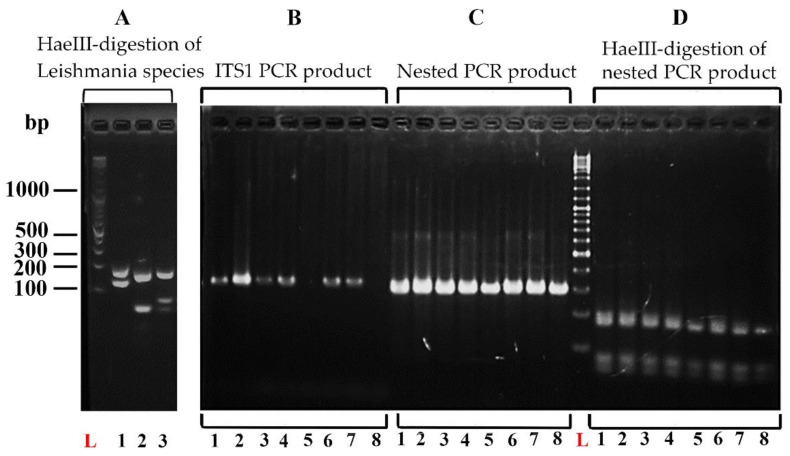
(**A**) RFLP patterns of reference strains (L: 100 bp DNA ladder, lane 1: *L. major*, 2: *L. tropica*, 3: *L. donovani*), identification of *Leishmania* DNA from clinical samples; (**B**) amplified by ITS1 PCR, (**C**) amplified by modified nested PCR, (**D**) RFLP analysis of the product of modified nested PCR.

**Figure 4:**
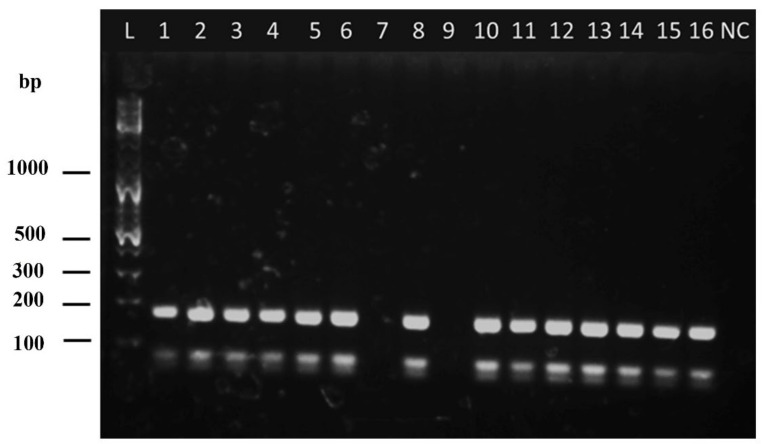
Agarose gel (2%) electrophoresis of the *Hae*III restriction enzyme digested product of modified nested ITS1 PCR (L: 100 bp DNA ladder, 1–16: clinical samples from the cohort of CL cases, NC: negative control).

Out of the 194 samples tested, the nested ITS1 PCR detected the parasite’s DNA in 183 (94.3%) samples. *Hae*III restriction digestion and RFLP analysis of *L. tropica, L. major*, and *L. donovani* DNA showed distinct restriction banding patterns that enable species identification (Figure 3). The RFLP analysis in the preliminary survey demonstrated a banding pattern corresponding to that of *L. donovani* in all the positive samples (Figure 3 and Figure 4). The PCR product and the fragment sizes of PCR products in base pairs (bp) generated by *Hae*III restriction digestion are shown in Table 2, along with the strains and GenBank accession numbers.

### 3.4. Diagnostic Accuracy of SSS against Nested ITS1 PCR 

The sensitivity and specificity of SSS in case detection, when compared against nested ITS1 PCR, were 80.9% and 81.8% in the present study. The positive predictive value of the SSS was high, at 98.7%, while the negative predictive value was very low, at 20.5%. 

### 3.5. Comparison of the Diagnostic Ability of Clinically Diagnosed Cases of CL in the Hospital Setting by Slit Skin Smear (SSS) and Modified Nested ITS1 PCR 

Case detection by SSS and modified nested ITS1 PCR was compared among the lesions that were detected as clinically confirmed and clinically doubtful by the dermatology specialist (Table 3).

In the clinically confirmed category, nested PCR detected 95% of the cases, whereas it was 79.4% by SSS. It was evident that approximately 20% of the lesions that were clinically diagnosed as CL were missed by SSS. In the doubtful category, SSS detected less than 60% of the cases, whereas nested PCR detected a significantly higher percentage compared to SSS (95%, *p* < 0.01). Nested PCR performed equally well in detecting confirmed and doubtful cases (*p* = 0.605). The ability of the SSS was significantly low in detecting cases in the doubtful category (*p* < 0.05).

### 3.6. Comparison of the Diagnostic Ability of Different Types of CL Lesions by Slit Skin Smear (SSS) and Modified Nested ITS1 PCR

**Figure 5:**
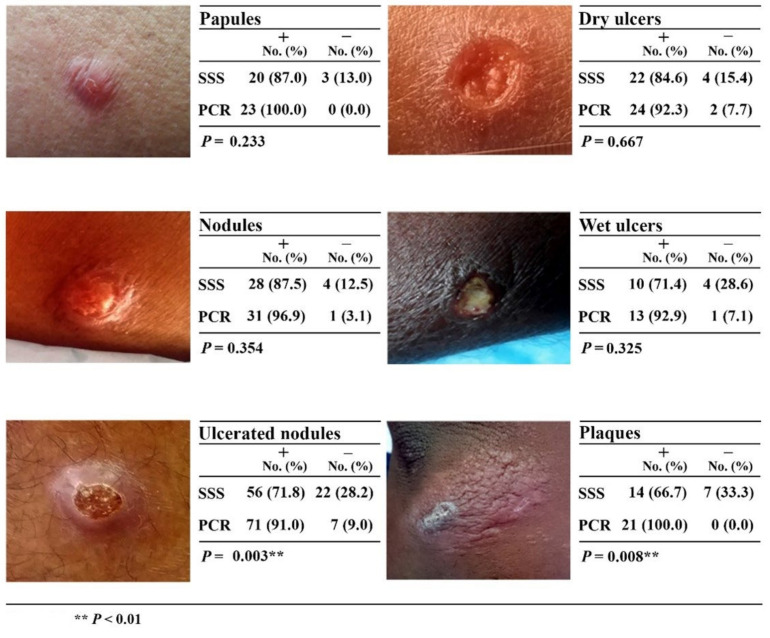
Diagnostic ability of SSS and nested PCR against the nature of the skin lesions: ** *p* < 0.01.

A higher percentage of papular (87%) and nodular (87.5%) types of lesions was diagnosed by SSS, whereas the detection of ulcerated nodules (71.8%; *p* < 0.01) and plaques (66.7%; *p* < 0.01) was significantly lower compared to PCR. Detection of parasites by nested ITS1 PCR was >90% among all types of lesions (Figure 5). Negative results by microscopy and nested PCR in different clinical lesions were significantly correlated (r2 = 0.782; *p* < 0.05).

## 4. Discussion

The present study successfully established a highly sensitive (94.3%) and specific modified nested ITS1 PCR-RFLP with novel inner primers for the diagnosis and species identification of CL in Sri Lanka. LITSR/L5.8S (Table 1) primers amplify a 320-base-pair fragment of the ITS1 region of *Leishmania* genus-specific DNA [25]. Amplification of the ITS region, which lies between the genes coding for small-subunit RNA and 5.8S RNA, allows the direct detection of parasite DNA from clinical samples [22,25]. In the present study, ITS1 PCR was used as the first PCR, and we have advanced the method into a nested PCR by using novel inner primers. ITS1, as well as the modified nested PCR, was sensitive enough to detect DNA directly from clinical samples, without the need for culturing the parasite, which is a slow and laborious process.

ITS1 PCR is already well established, and in our study, ITS1 PCR amplifying *Leishmania* genus-specific DNA using LITSR/L5.8S primers was sensitive enough to detect as little as 25 fg of parasite DNA (Figure 2). In an earlier study conducted in Sri Lanka, the same primers of ITS1 PCR detected up to 10 fg of parasite DNA [19]. Differences in DNA extraction and PCR conditions probably accounted for the variability. However, in our study, when the ITS1 product was re-amplified in the nested PCR using the novel inner primers (LITSR-inner and L5.8S-inner), sensitivity was improved by 10-fold. The amount of DNA detected was 2.5 fg, which was at 10-2 in the serial dilutions. However, serial dilutions were performed only up to the concentration of 10-2. The trace amount of DNA at 10-2 in the ITS1 PCR, which was insufficient to be amplified by the primers of ITS1 PCR, was sufficient as a template for the novel primers of the nested PCR, which amplified it, giving a band of the same intensity (Figure 2). Therefore, we could extrapolate that the modified nested PCR possibly has higher sensitivity than estimated. Earlier studies on ITS1 PCR have also demonstrated higher sensitivity when a nested ITS1-PCR is carried out [26]. This fact was one of the reasons that we deployed a nested PCR in our study. Any non-specific amplification in the first round will not be amplified in the second round, and the second set of primers amplify regions internal to the regions of the first PCR, making the method more specific than the direct PCR [26,27,28]. The major disadvantage of nested PCR is that there is a risk of contamination due to the carryover of products between the two reactions [29]. Precautions to avoid carryover contaminations are important, and in the present study, strict precautions such as aliquoted reagents, dedicated bench space, dedicated pipettes and laboratory equipment, sterilized pipette tips, avoidance of pipette tip contact with surfaces or insides of tubes, precautions when opening caps of PCR tubes, frequent hand washing, use of disposable gloves, and bench cleaning were followed. Being more labor-intensive and time-consuming are further limitations. 

In an earlier study, Mo-STNPCR as a single-tube reaction was shown to be 100% sensitive for the diagnosis of CL in Sri Lanka [20]. Although carryover contamination is avoided, the tendency to form non-specific bands is a disadvantage of single-tube reactions due to the mixing of two primer pairs in the same solution [30]. The modified nested PCR in the present study confirmed more than 94% of cases in the cohort that we studied (*n* = 194). Differences in sensitivity among assays can arise due to differences in PCR conditions and DNA extraction procedures, as well as due to the genetic diversity of the parasite. ITS1 is a highly conserved region; however, the associated polymorphisms allow differentiation between species [29]. The genetic heterogeneity of *L. donovani* in Sri Lanka is evident through many factors (listed), which can contribute to the differing sensitivity between diagnostic techniques: (i) genomic analysis of *L. donovani* isolates of global distribution showed that the isolates fell into five large groups based on geographic origin but showed vast genetic variation within these groups (this study included Sri Lankan isolates) [30]; (ii) sub-phenotypic variation in classical CL caused by *L. donovani* was evident between the northern and southern foci of disease transmission in Sri Lanka, suggesting strain differences [31]; (iii) high haplotype diversity, with sub-clusters showing clinically relevant characteristics [32]; (iv) emergence of treatment failure and non-responsiveness to standard treatment [17,33], which is multifactorial in origin, but genetic variations contribute [34].

The described nested ITS1 PCR is a *Leishmania* genus-specific assay and the primer pairs will detect different species, which can then be distinguished by RFLP analysis. Our study confirmed that the only parasite causing CL among the cohort of 194 patients from the southern focus was *L. donovani*, which is comparable with the conclusion of an earlier study that stated that *L. donovani* is the only species causing CL in Sri Lanka [19]. Although RFLP analyses have been shown to identify hybrid strains of parasites in other endemic settings, the RFLP pattern observed was the same across the present study, and no strain differences were observed [35]. However, the foreign travel of Sri Lankans has increased immensely over the years and also the influx of foreigners in the form of leisure or labor has also risen. Labor influx has been significant from India, where a high burden of leishmaniasis is reported. As such, we are at constant risk of importing new parasites. Supporting this fact, the early stage of leishmaniasis in the country was characterized by imported disease [36], while the genetic homology of the local parasite to *L. tropica* and *L. major* suggests hybridization and probable origin and importation from East Africa, which further reiterates the importance of importation [13]. Furthermore, the emergence of atypical clinical presentations and drug resistance also necessitates surveillance of the existence of other *Leishmania* species in Sri Lanka. It will be useful to screen atypical presentations and people with travel history. RFLP analysis offers a more convenient means of species identification as it is only one step further from PCR, simple, rapid, and gives accurate identification of the parasite to the species level. PCR-RFLP can be performed as a single procedure for the detection and identification of *Leishmania* species from clinical samples in laboratory settings with adequate facilities. Cost is a limiting factor for regular use in resource-poor settings, but PCR-RFLP is less laborious and is low-cost compared to isoenzyme typing or MLEE [26] for species identification. PCR-RFLP analysis by itself is unable to differentiate between the members of the *L. donovani* complex [37]. At present, only *L. donovani* is known to be the causative agent of CL in Sri Lanka, and no case of *L. infantum* infection is reported [19]. In addition, we performed sequence analysis on 10 randomly chosen 10 samples, and all were confirmed to be *L. donovani* (data not presented). PCR-RFLP of the cysteine protease B (*cpb*) gene is reported to differentiate these two species [38] and we are planning to incorporate this technique in our future studies.

Nucleic acid-based techniques are expensive and remain limited to reference settings in Sri Lanka, but they are still important to detect challenging cases. The present study demonstrated that the routine diagnostic method was missing more than 20% of the clinically diagnosed cases of CL and that the diagnosis of clinically doubtful lesions using SSS was challenging (<60%). Furthermore, SSS showed lower performance in certain lesion types (Figure 5) and in chronic lesions where parasite density is low (data not shown). However, nested ITS1 PCR performed exceedingly well in the diagnosis of all these categories of lesions, including chronic lesions, which demonstrates the importance of making highly sensitive tests available. Missed cases contribute to the onward transmission of the disease in an era where the diagnosis and treatment of every case has become vital in the absence of a separate control program in Sri Lanka. Hence, we sought to develop techniques of higher sensitivity to aid in disease control at a time when the disease burden continues to rise and identifying and treating every case has become important. We were able to achieve 10 times higher sensitivity when combined with a nested step than ITS1 PCR alone in the present study (detection level 25 fg vs. 2.5 fg). Additionally, the high specificity of the nested PCR technique is indispensable for settings such as Sri Lanka, where other infectious diseases, such as leprosy, cutaneous tuberculosis, and fungal lesions, are mimicking CL. Before committing patients to toxic treatment regimens, it is important to confirm the diagnosis, which is supported by the high specificity of nested PCR. 

In the present study, 11 samples were detected as negative by the modified nested PCR. Two among them were determined as false negatives (18.2%) as they were positive by SSS. The sampling site within the lesion is known to influence the diagnostic techniques due to the non-uniform distribution of parasites within the skin lesion [39]. The focal existence of parasites within skin lesions was demonstrated in histopathological studies, suggesting that multiple samples from different sites of the lesion are required for better efficiency; however, this is not ethically sound [40]. These differences may influence the discrepancy in results obtained by SSS and nested PCR, which may be more pronounced in lesions with ultra-low parasite densities. The quality of the sample obtained for nested PCR may also play a role because the *Leishmania* parasites are within macrophages in the dermis and superficial punches involving the epidermis could lead to false negatives. Moreover, misidentification of artefacts as amastigotes, especially extracellular ones, by SSS might lead to false positive results in which PCR could not amplify the parasite DNA. 

The requirement of invasive tissue samples is a disadvantage of PCR. It limits the use of PCR in lesions in sensitive areas, such as closer to the eye or pinna, where the collection of a punch biopsy specimen is difficult. In the present study, almost every punch biopsy site tended to bleed and subsequently required suturing. The procedure is cumbersome to the patients as the wound requires follow-up care with cleaning, dressing, and suture removal. It also increases the risk of infection. In the present study, strict aseptic measures were followed to minimize the risk of infection. The less invasive sampling option of spotting scraped lesion material on FTA cards (Whatman^®^) is available for PCR studies and overcomes the difficulties of collecting punch biopsies [41]. Sample collection on FTA cards is also field-friendly and allows easy storage. They can be easily sent by post to a reference facility for diagnostic purposes. 

## 5. Conclusions

In conclusion, the present study has established a highly sensitive and specific nested ITS1 PCR-RFLP, which could detect up to 2.55 fg of parasite DNA and demonstrated that it can be successfully used for the diagnosis and speciation of *Leishmania donovani* causing CL in Sri Lanka. Although the cost and requirement of laboratory facilities are limiting factors, such tests will be useful, especially as a second-line diagnostic technique in diagnosing difficult or missed cases (by routine diagnostic method/SSS), cases with atypical clinical presentations, foreign travel, drug resistance, and chronic lesions. Paired with RFLP, it will provide a surveillance tool for the epidemiological monitoring of the emergence of new parasites. Making highly sensitive tests available is imperative for case detection and control. 

## Figures and Tables

**Table 1:** ITS1 and modified nested PCR primer sequences.

Primer *	Sequence
**Outer primers**	
LITSR	5′-CTGGATCATTTTCCGATG-3′
L5.8S	5′-TGATACCACTTATCGCACTT-3′
**Inner primers**	
LITSR inner	5′-CATTTTCCGATGATTACACC-3′
L5.8S inner	5′-TACTGCGTTCTTCAACGA-3′

* The outer primers are utilized in the ITS1 region amplification in the already established ITS1 PCR [25]. The two inner primers, LITSR-inner and L5.8S-inner, are two novel primers used for the first time in the modified nested PCR carried out as a part of this study.

**Table 2:** Fragment size of *Leishmania* ITS genes generated by digestion with *Hae*III.

*Leishmania* Species and Strain	GenBank Accession Number	PCR Product Size (bp)	Restriction Fragment Sizes (bp)
*L. major*—5ASKH	KU680845	312	124, 188
*L. tropica*—OD	EU326226	291	20, 48, 55, 168
*L. donovani*—DD8	MH202977	293	55, 69, 169

**Table 3:** Detection of clinical cases of CL by SSS and nested PCR.

	Clinical Diagnosis				
	Confirmed	Doubtful			
	Positive (%)	Negative (%)	Positive (%)	Negative (%)	*p*
Nested PCR	166 (94.9)	9 (5.1)	17 (89.5)	2 (10.5)	0.605
SSS	139 (79.4)	36 (20.6)	11 (57.9)	8 (42.1)	0.044

## Data Availability

Data contained within the article.

## References

[1] World Health Organization Leishmaniasis. https://www.who.int/news-room/fact-sheets/detail/leishmaniasis.

[2] Mcgwire B.S., Satoskar A.J. (2014). Leishmaniasis: Clinical syndromes and treatment. QJM-Int. J. Med..

[3] Anon (2008). General Circular no. 02/102/2008.

[4] Karunaweera N.D., Ginige S., Senanayake S., Silva H., Manamperi N., Samaranayake N., Siriwardana Y., Gamage D., Senerath U., Zhou G. (2020). Spatial Epidemiologic Trends and Hotspots of Leishmaniasis, Sri Lanka, 2001–2018. Emerg. Infect. Dis..

[5] Nawaratna S.S., Weilgama D.J., Rajapaksha K. (2009). Cutaneous leishmaniasis in Sri Lanka: A study of possible animal reservoirs. Int. J. Infect. Dis..

[6] Rosypal A., Tripp S., Kinlaw C., Hailemariam S., Tidwell R., Lindsay D., Rajapakse R., Sreekumar C., Dubey J. (2010). Surveillance for antibodies to *Leishmania* spp. in dogs from Sri Lanka. J. Parasitol..

[7] Abayaweera C.A., Siriwardana Y., Abeywardana T., Rathnayaka R., Kumarasinghe H., Karunaweera N.D. Dogs as a possible animal reservoir for leishmaniasis in Dickwella, Sri Lanka. Proceedings of the Annual Research Procedings University of Colombo.

[8] Iddawela D., Vithana S.M.P., Atapattu D., Wijekoon L. (2018). Clinical and epidemiological characteristics of cutaneous leishmaniasis in Sri Lanka. BMC Infect. Dis..

[9] Rajakaruna R.S., Jayathilake P., Wijerathna H., Fernando A., Ginarathne K., Naullage N., Silva S., Thananjayan K., Amarasiri L., Jayasundara N. (2021). Canine Vector-Borne Diseases of Working Dogs of the Sri Lanka Air Force, Free-Roaming, and Privately-Owned Dogs. Res. Square.

[10] Siriwardana H., Chandrawansa P., Sirimanna G., Karunaweera N. (2012). Leishmaniasis in Sri Lanka: A decade old story. Sri Lankan J. Infect. Dis..

[11] Amarasinghe A., Wickramasinghe S.J.A.P. (2020). A comprehensive review of cutaneous leishmaniasis in Sri Lanka and identification of existing knowledge gaps. Acta Parasitol..

[12] Karunaweera N., Pratlong F., Siriwardane H., Ihalamulla R., Dedet J. (2003). Sri Lankan cutaneous leishmaniasis is caused by Leishmania donovani zymodeme MON-37. Trans. R. Soc. Trop. Med. Hyg..

[13] Lypaczewski P., Matlashewski G.J.T.L.M. (2021). Leishmania donovani hybridisation and introgression in nature: A comparative genomic investigation. Lancet Microbe.

[14] Rajapaksa U., Ihalamulla R., Karunaweera N. (2005). First report of mucosal tissue localisation of leishmaniasis in Sri Lanka. Ceylon Med. J..

[15] Ranasinghe S., Zhang W.-W., Wickremasinghe R., Abeygunasekera P., Chandrasekharan V., Athauda S., Mendis S., Hulangamuwa S., Matlashewski G., Pratlong F.J.P. (2012). Leishmania donovani zymodeme MON-37 isolated from an autochthonous visceral leishmaniasis patient in Sri Lanka. Pathog. Glob. Health.

[16] Siriwardana Y., Deepachandi B., Gunasekara C., Warnasooriya W., Karunaweera N.D. (2019). Leishmania donovani Induced Cutaneous Leishmaniasis: An Insight into Atypical Clinical Variants in Sri Lanka. J. Trop. Med..

[17] Silva H., Liyanage A., Deerasinghe T., Chandrasekara V., Chellappan K., Karunaweera N.D. (2021). Treatment failure to sodium stibogluconate in cutaneous leishmaniasis: A challenge to infection control and disease elimination. PLoS ONE.

[18] Giantsis I.A., Chaskopoulou A., Claude Bon M.J. (2017). Direct multiplex PCR (dmPCR) for the identification of six phlebotomine sand fly species (Diptera: Psychodidae), including major Leishmania vectors of the Mediterranean. J. Econ. Entomol..

[19] Ranasinghe S., Wickremasinghe R., Hulangamuwa S., Sirimanna G., Opathella N., Maingon R.D., Chandrasekharan V. (2015). Polymerase chain reaction detection of LeishmaniaDNA in skin biopsy samples in Sri Lanka where the causative agent of cutaneous leishmaniasis is Leishmania donovani. Mem. Inst. Oswaldo Cruz.

[20] Deepachandi B., Weerasinghe S., Soysa P., Karunaweera N., Siriwardana Y. (2019). A highly sensitive modified nested PCR to enhance case detection in leishmaniasis. BMC Infect. Dis..

[21] Kothalawala H., Karunaweera N. (2016). Loop-mediated isothermal amplification assay as a sensitive diagnostic tool for Leishmania donovani infections in Sri Lanka. Ceylon Med. J..

[22] Cupolillo E., Grimaldi Jr. G., Momen H., Beverley S.M. (1995). Intergenic region typing (IRT): A rapid molecular approach to the characterization and evolution of Leishmania. Mol. Biochem. Parasitol..

[23] Schönian G., Schnur L., El Fari M., Oskam L., Kolesnikov A.A., Sokolowska-Köhler W., Presber W. (2001). Genetic heterogeneity in the species Leishmania tropica revealed by different PCR-based methods. Trans. R. Soc. Trop. Med. Hyg..

[24] Koarashi Y., Cáceres A.G., Saca F.M.Z., Flores E.E.P., Trujillo A.C., Alvares J.L.A., Yoshimatsu K., Arikawa J., Katakura K., Hashiguchi Y. (2016). Identification of causative Leishmania species in Giemsa-stained smears prepared from patients with cutaneous leishmaniasis in Peru using PCR-RFLP. Acta Trop..

[25] El Tai N., Osman O., El Fari M., Presber W., Schönian G. (2000). Genetic heterogeneity of ribosomal internal transcribed spacer in clinical samples of Leishmania donovani spotted on filter paper as revealed by single-strand conformation polymorphisms and sequencing. Trans. R. Soc. Trop. Med. Hyg..

[26] Schönian G., Nasereddin A., Dinse N., Schweynoch C., Schallig H.D., Presber W., Jaffe C.L. (2003). PCR diagnosis and characterization of Leishmania in local and imported clinical samples. Diagn. Microbiol. Infect. Dis..

[27] Cruz I., Canavate C., Rubio J., Morales M., Chicharro C., Laguna F., Jimenez-Mejias M., Sirera G., Videla S., Alvar J. (2002). A nested polymerase chain reaction (Ln-PCR) for diagnosing and monitoring Leishmania infantum infection in patients co-infected with human immunodeficiency virus. Trans. R. Soc..

[28] da Silva M.A.L., Soares C.R.P., Medeiros R.A., Medeiros Z., de Melo F.L. (2013). Optimization of single-tube nested PCR for the diagnosis of visceral leishmaniasis. Exp. Parasitol..

[29] Akhoundi M., Downing T., Votýpka J., Kuhls K., Lukeš J., Cannet A., Ravel C., Marty P., Delaunay P., Kasbari M. (2017). Leishmania infections: Molecular targets and diagnosis. Mol. Asp. Med..

[30] Franssen S.U., Durrant C., Stark O., Moser B., Downing T., Imamura H., Dujardin J.-C., Sanders M.J., Mauricio I., Miles M.A.J.E. (2020). Global genome diversity of the Leishmania donovani complex. eLife.

[31] Siriwardana Y., Deepachandi B., Weerasinghe S., Karunaweera N., Udagedara C., Warnasuriya W., Ranawaka R.R., Kahawita I. (2021). First Evidence from Sri Lanka for Subphenotypic Diversity within L. donovani-Induced Classical Cutaneous Leishmaniasis. Biomed Res. Int..

[32] Kariyawasam U.L., Selvapandiyan A., Rai K., Wani T.H., Ahuja K., Beg M.A., Premathilake H.U., Bhattarai N.R., Siriwardena Y.D., Zhong D. (2017). Genetic diversity of Leishmania donovani that causes cutaneous leishmaniasis in Sri Lanka: A cross sectional study with regional comparisons. BMC Infect. Dis..

[33] Refai F.W., Madarasingha N.P., Fernandopulle R., Karunaweera N. (2016). Nonresponsiveness to standard treatment in cutaneous leishmaniasis: A case series from Sri Lanka. Trop. Parasitol..

[34] Vergnes B., Gourbal B., Girard I., Sundar S., Drummelsmith J., Ouellette M.J.M., Proteomics C. (2007). A proteomics screen implicates HSP83 and a small kinetoplastid calpain-related protein in drug resistance in Leishmania donovani clinical field isolates by modulating drug-induced programmed cell death. Mol. Cell. Proteom..

[35] Kato H., Gomez E.A., Seki C., Furumoto H., Martini-Robles L., Muzzio J., Calvopiña M., Velez L., Kubo M., Tabbabi A. (2019). PCR-RFLP analyses of Leishmania species causing cutaneous and mucocutaneous leishmaniasis revealed distribution of genetically complex strains with hybrid and mito-nuclear discordance in Ecuador. PLoS Negl. Trop. Dis..

[36] Naotunne T., Rajakulendran S., Abeywickreme W., Kulasiri C., Perera J., Premaratne U., Attygalle D., Mendis K. (1990). Cutaneous leishmaniasis in Sri Lanka. An imported disease linked to the Middle East and African employment boom. Trop. Geogr. Med..

[37] Fotakis E.A., Giantsis I.A., Avgerinou A., Kourtidis S., Agathaggelidou E., Kapoula C., Dadakou G., Vontas J., Chaskopoulou A. (2019). Identification of Leishmania species in naturally infected sand flies from refugee camps, Greece. Emerg. Infect. Dis..

[38] Hide M., Banuls A.-L. (2006). Species-specific PCR assay for L. infantum/L. donovani discrimination. Acta Trop..

[39] Ramírez J.R., Agudelo S., Muskus C., Alzate J.F., Berberich C., Barker D., Velez I.D. (2000). Diagnosis of cutaneous leishmaniasis in Colombia: The sampling site within lesions influences the sensitivity of parasitologic diagnosis. J. Clin. Microbiol..

[40] Sharquie K.E., Hassen A.S., Hassan S.A., Al-Hamami I.A. (2002). Evaluation of diagnosis of cutaneous leishmaniasis by direct smear, culture and histopathology. Saudi Med. J..

[41] Kato H., Watanabe J., Nieto I.M., Korenaga M., Hashiguchi Y. (2011). Leishmania species identification using FTA card sampling directly from patients’ cutaneous lesions in the state of Lara, Venezuela. Trans. R. Soc. Trop. Med. Hyg..

